# The Cost of Provisions in Institutions.—XII

**Published:** 1904-07-16

**Authors:** 


### HOSPITAL ADMINISTRATION.

### CONSTRUCTION AND ECONOMICS.

### THE COST OF PROVISIONS IN INSTITUTIONS.—XII.

BY THE AUTHOR OF "HOSPITAL EXPENDITURE—THE COMMISSARIAT."

(*Continued from page* 267.)

#### THE SECRET OF CONTROL.

ECONOMY in victualling a household depends, as we have seen, first on the limitation of numbers, secondly on skilled buying, and thirdly on effective control of the supplies. The first is not always possible or desirable. Indeed the tendency of large institutions of late years has been more and more to draw the staff within the walls, and limit the amount of outside service.

With regard to the second point, the question of prices and quality, we shall have something to say later on. The third point, effective control of the supplies, is the real crux of the position, and it is in this direction that many hospital managers feel themselves hopelessly at a loss.

There are two ways in which control can be secured. Place in charge of the household an energetic, watchful, and conscientious manager, whether man or woman, filled with zeal for the well-being of the charity, and endowed with tact to guide all subordinates in the way in which they should go, and you will have the best possible results, both as regards efficiency and economy. But alas, these heaven-sent managers are rare. Every hospital can point in its annals to some golden age when everything went well as if by magic, and the bills were a source of pleasure to the committee. These epochs do not endure, nor can those who produce these happy results transmit the secret of their power to their successors. No institution can make sure, however diligently it may search, of securing the ideal House Keeper. It is safer to rely on a perfected system of accounts.

It will not for one moment be contended that a mechanical method of management can produce in the household the same contented spirit of organised co-operation which the supervision of a good matron or superintendent infuses. But a perfect system of accounts is the best of all coadjutors to the person who holds this responsible post, and when, as so constantly happens, there is no person in the institution who possesses this rare gift of government, a perfect system of accounts will be found quite unobtrusively to fill the vacant place in enforcing the spirit of economy. The aim of a perfect system of accounts is to bring responsibility home direct to everyone concerned in using the supplies.

The complex character of the hospital household renders economy very difficult indeed, unless separate account be taken of the supplies for each section composing it. This is now recognised in many of the best-managed institutions, as our readers will have seen from a study of the preceding articles of this series. It is no part of our scheme to set out in this place a detailed system of accounts; we may refer all who are interested in the matter to the recently published new edition of "Burdett's Uniform System of Accounts for Hospitals and Institutions." *

The difficulty of keeping a separate account of the provisions used respectively for the patients, the officers' table, the nurses' table, and the household, is more imaginary than real, and will be found to originate not with the secretarial department, but with the housekeeper. We shall be told that it is more economical, especially in moderate-sized institutions, for the different sections of the household to "work in" together, so that joints from the officers' table can be finished by the servants, and so forth. If any advantage is to be gained by this system, and of this we are somewhat doubtful, it need not in practice hinder the strict subdivision of the supplies. Almost the only article of provisions which it may be convenient to transfer from one table to another is meat, and an experienced housekeeper should have no difficulty whatever in estimating the proportion of such transferred joints which has fallen to the share of the second table.

It may be assumed that returns stating the amount required of every article of provisions, for every section of the household, are indispensable to any large institution. No matter in what form they are rendered, or whether the class of persons is specified for whom such provisions are needed, some such daily reckoning there must be, for without this the machine could not work at all. To get a complete grasp then of the cost of the commissariat department, it will not be necessary to introduce any elaborate novelties. All that is required is so to arrange these daily estimates of household consumption, that they may afford the necessary data, from which a weekly return of the expenditure on all the different sections of the household may be compiled.

There are a great many institutions in which all the information necessary for ascertaining the weekly cost of boarding the patients, medical officers, nurses, and household, lie buried in the housekeeper's or steward's book, and never see the light. There are very few, indeed, who realise the importance of combining these humble details into a return readily comprehensible by the weekly board of governors to which it is submitted.

It would be a surprise to most managers if they could realise the constant fluctuations in cost which such a system conscientiously worked out can reveal. It is not too much to say that it would definitely and once for all put a stop to any serious waste of provisions. As for instance, let it be supposed that the larder accommodation is bad, and that the meat "turns off" rapidly in hot weather. In an institution where things are allowed to drift, the sacrifice of joint after joint of cold meat, as soon as the thermometer rises, will be regarded merely as "such a pity." Under the perfected system of accounts, such misadventures would send up the cost of provisions in any week of hot weather to an extent which would instantly draw attention to the loss incurred by the hospital on account of its defective accommodation. The expense of improving the larders would thus clearly commend itself to the managers as a lesser evil than that of continual waste.

Such instances might be almost indefinitely multiplied. In provincial institutions, where the baking is often very bad indeed, large quantities of bread are wasted when it is

* "Uniform System of Accounts for Hospitals and Public Institutions, with special forms of account, complete sets of books, certain suggested checks upon expenditure, forms of tender, and other aids to economy, together with an index of classification whereby every item may be dealt with under identical heads by every group of institutions." (The Scientific Press Ltd.)

sent in too sour for use. If this were only reflected in the weekly returns of the cost of the household, the contractor would speedily be brought to order and compelled to take back any inferior bread supplied. But, apart from the checking of such abuses as we have indicated, the knowledge that the catering is done under the immediate eye of the governors has a wonderfully stimulating effect upon housekeeper or steward, and the weekly interest of hearing how the returns work out has a marked effect in keeping everyone concerned on the alert.

We propose, in our next article, to give a few specimen forms, by aid of which such returns may be prepared.

